# A Fly’s Eye View of Natural and Drug Reward

**DOI:** 10.3389/fphys.2018.00407

**Published:** 2018-04-18

**Authors:** Eve G. Lowenstein, Norma A. Velazquez-Ulloa

**Affiliations:** Department of Biology, Lewis & Clark College, Portland, OR, United States

**Keywords:** *Drosophila*, natural reward, drug reward, ethanol, nicotine, cocaine, amphetamine, methamphetamine

## Abstract

Animals encounter multiple stimuli each day. Some of these stimuli are innately appetitive or aversive, while others are assigned valence based on experience. Drugs like ethanol can elicit aversion in the short term and attraction in the long term. The reward system encodes the predictive value for different stimuli, mediating anticipation for attractive or punishing stimuli and driving animal behavior to approach or avoid conditioned stimuli. The neurochemistry and neurocircuitry of the reward system is partly evolutionarily conserved. In both vertebrates and invertebrates, including *Drosophila melanogaster*, dopamine is at the center of a network of neurotransmitters and neuromodulators acting in concert to encode rewards. Behavioral assays in *D. melanogaster* have become increasingly sophisticated, allowing more direct comparison with mammalian research. Moreover, recent evidence has established the functional modularity of the reward neural circuits in *Drosophila*. This functional modularity resembles the organization of reward circuits in mammals. The powerful genetic and molecular tools for *D. melanogaster* allow characterization and manipulation at the single-cell level. These tools are being used to construct a detailed map of the neural circuits mediating specific rewarding stimuli and have allowed for the identification of multiple genes and molecular pathways that mediate the effects of reinforcing stimuli, including their rewarding effects. This report provides an overview of the research on natural and drug reward in *D. melanogaster*, including natural rewards such as sugar and other food nutrients, and drug rewards including ethanol, cocaine, amphetamine, methamphetamine, and nicotine. We focused mainly on the known genetic and neural mechanisms underlying appetitive reward for sugar and reward for ethanol. We also include genes, molecular pathways, and neural circuits that have been identified using assays that test the palatability of the rewarding stimulus, the preference for the rewarding stimulus, or other effects of the stimulus that indicate how it can modify behavior. Commonalities between mechanisms of natural and drug reward are highlighted and future directions are presented, putting forward questions best suited for research using *D. melanogaster* as a model organism.

## Introduction

Animals need to distinguish beneficial stimuli in order to survive. There is partial conservation among reward systems across species (Scaplen and Kaun, 2016). Mammalian models of reward have allowed for the dissection of the several components of reward as well as the mapping of these components for different neural circuits and neurotransmitters. Further dissection of reward circuits using large-scale genetic screens could help to elucidate the genetic and molecular mechanisms of reward. For this complementary approach, the *Drosophila melanogaster* model system is ideally suited and allows for targeted genetic manipulations, which are necessary to determine causality for the identified genetic factors (Venken and Bellen, 2014). *Drosophila* also allows for targeted genetic manipulations, which are necessary to determine causality.

*Drosophila* has been a primary model organism for elucidating the role of genes and identifying molecular mechanisms and neural circuits underlying biological processes (Rubin and Lewis, 2000 ; Bellen et al., 2010 ; Venken et al., 2011). The whole genome of the fly has been sequenced and annotated. It is believed that between 65 and 75% of human disease-causing genes have a functional homolog in *Drosophila* (Reiter et al., 2001 ; Chien et al., 2002 ; Yamamoto et al., 2014). The fast life cycle, high fecundity, smaller space needed to maintain colonies, lower cost of fly husbandry, and the wide array of commercially available transgenic fruit fly strains make the fruit fly a great model organism for forward and reverse genetic screens as well as genomic approaches for identification and rapid validation of genes involved in reward. Knowledge gathered about *Drosophila* has been compiled in databases that specialize in different content (modENCODE, Celniker et al., 2009 ; FlyAtlas, Chintapalli et al., 2007 ; FlyBase, Gramates et al., 2017 ; Larval Olympiad data set and FlyEM, HHMI Janelia Research Campus; Virtual Fly Brain, Milyaev et al., 2012 ; Flybrain Neuron Database, The University of Tokyo). *Drosophila* labs and public institutions across the world develop and maintain collections of mutants and transgenic tools that allow for probing the function of nearly every gene in the fly with exquisite spatial and temporal control, including single cell resolution and restriction of the manipulation to specific developmental stages or segments of a behavioral task (Brand and Perrimon, 1993 ; Jenett et al., 2012 ; Venken and Bellen, 2014).

The possibility of altering gene expression or controlling neuronal activity at the single-cell level makes flies an ideal model to dissect reward circuits and allows for mapping how specific genetic networks act within specific cells in a neuronal circuit. These tools have allowed for the mapping of genes and neuronal circuits that control natural and drug reward, revealing similarities in the organization of the reward system in mammals and *Drosophila*, including the role of DA and the general rules for reward processing (de Araujo, 2016 ; Scaplen and Kaun, 2016).

Behavioral assays have been developed to study natural and drug reward in *D. melanogaster* (Kaun et al., 2012). The behavioral assays for studying reward vary between mammals and insects. Mammalian assays of natural reward regularly involve operant conditioning, such as pressing a lever, and are dependent on a specific action by the animal to obtain an US (Perry and Barron, 2013). In contrast, the paradigms used to study insect reward involve presentation of the CS and US by the researcher, and are thus independent of the action of the animals (Perry and Barron, 2013). The study of reward in *D. melanogaster* has largely focused on classical conditioning learning paradigms using either innately rewarding or punishing US paired with a CS. Learning is said to take place based on the response to the CS after training. Assays to test other aspects of the reinforcing stimuli involve voluntary consumption and two-choice preference assays. These assays provide a broad picture of reinforcing stimuli, ultimately determining whether these stimuli have reinforcing properties for short and/or long-term memories. Studies have probed multiple aspects of natural reward across developmental stages, including appetitive and aversive stimuli in larval and adult stage *Drosophila* (Diegelmann et al., 2013 ; Perry and Barron, 2013 ; Davis, 2015 ; Landayan and Wolf, 2015 ; Das et al., 2016 ; Scaplen and Kaun, 2016 ; Kaun and Rothenfluh, 2017 ; Cognigni et al., 2018).

Only recently has the study of drug reward in *Drosophila* used similar assays to those used to dissect natural reward (Kaun et al., 2012). Palatability, voluntary consumption, and conditioned odor preference behavior assays have identified genes, molecular pathways, and neural circuits underlying drug reward, and have demonstrated that certain drugs, such as ethanol, can be rewarding for flies. Much more is known about the acute effects of drugs in *Drosophila*. However, these acute drug assays do not focus on reward, but on the locomotor effects of the drugs. Nonetheless, some of the genes and neural circuits that have been identified with acute drug exposure match those underlying feeding (Landayan and Wolf, 2015). This suggests common factors in the mechanisms underlying drug and natural reward.

Recent reviews on this topic have focused on reward processing and the similarities between the *Drosophila* and mammalian reward systems (de Araujo, 2016 ; Scaplen and Kaun, 2016 ; Kaun and Rothenfluh, 2017 ; Cognigni et al., 2018). Research studying reward in *Drosophila* larva has been reviewed by Diegelmann et al. (2013). A comprehensive review by Das et al. (2016) focuses on food reward in *Drosophila*. Another recent review highlights the neurotransmitters and neural circuits that mediate both feeding and drug effects (Landayan and Wolf, 2015).

In this review, we have compiled information about the mechanisms underlying natural and drug reward in *Drosophila* organizing information according to the behavior elicited by the natural or drug stimulus. We focus on appetitive behaviors that indicate that a given stimulus is palatable, preferred when given a choice, and serves a reinforcement in a learning and/or memory assay, indicating its rewarding value. These behaviors in combination provide a view of the underlying mechanisms of reward from perception to reinforcement. We limit this review to three assays for natural rewards: palatability, assessed by the proboscis extension reflex response; preference, assessed in a choice assay; and reward, assessed in a conditioned odor preference assay. The natural stimuli included in this review are sugars, proteins, fatty acids, and water. The drugs included are ethanol, cocaine, amphetamine/methamphetamine, and nicotine. Most research on drug reward has focused on ethanol, for which studies about its palatability, preference, and reward have been conducted. Ethanol and other drugs have also been examined by looking at their locomotor effects. We included these, as neural circuits and genes that mediate locomotor drug effects show partial overlap with those of natural and ethanol reward (Landayan and Wolf, 2015 ; this review). The figures in this review highlight common factors and are meant to help identify gaps in knowledge.

## Natural Reward

Rewarding stimuli are attractive, eliciting a subjective degree of pleasure, a hedonic value, or ‘liking’ (Berridge and Kringelbach, 2008). In mammals, ‘liking’ is identified by studying facial expressions (Steiner et al., 2001 ; Berridge and Kringelbach, 2008 ; Berridge and Robinson, 2016). ‘Liking’ in mammals is mediated by opioid, endocannabinoid, and GABA-benzodiazepine signaling, and is localized to hedonic hot spots distributed throughout the limbic system (Berridge et al., 2009). Rewards also have incentive salience, evoking a strong desire or craving for the reward or ‘wanting’ in mammals this is largely mediated by the mesocorticolimbic system, with DA as the main neurotransmitter (Berridge et al., 2009 ; Berridge and Robinson, 2016).

‘Wanting’ in insects can be assessed by focusing on approach and consummatory responses in instrumental learning paradigms (Perry and Barron, 2013). Natural rewards have intrinsic incentive salience and can be used as unconditioned stimuli (US). Incentive salience can also apply to Pavlovian conditioned stimuli (CS), which are learned stimuli that are originally neutral but become predictors of reward through stimulus-stimulus association (Berridge et al., 2009).

A different way to dissect reward is to focus on how rewarding stimuli affect the activity of the neural circuits underlying both approach and consummatory behavior, from perception to motor function. The first neural circuits to be activated by either rewarding or punishing stimuli are the sensory systems. Rewarding stimuli are salient, and hence activate the areas of the nervous system that encode attention in the brain. Learned rewards activate learning and memory circuits. Lastly, rewarding stimuli elicit behavior, which is directed by activation of motor circuits (Schultz, 2015).

Below, we present an overview of the mechanisms involved in palatability, preference and reinforcement elicited by the following natural food stimuli: sugar, protein, fatty acid, and water. We focus on sugar reward and how sweet taste versus nutritive value are encoded. We organize the information by assay, as each assay probes different aspects of the rewarding stimulus. Palatability focuses on the initial perception of the stimulus. Consumption and preference are tested using a two-choice assay to study consummatory behavior. Lastly, conditioned odor preference reflects the reinforcing properties of the stimulus.

### Sugar Palatability and Preference

#### Palatability: Proboscis Extension Reflex

**FIGURE 1:**
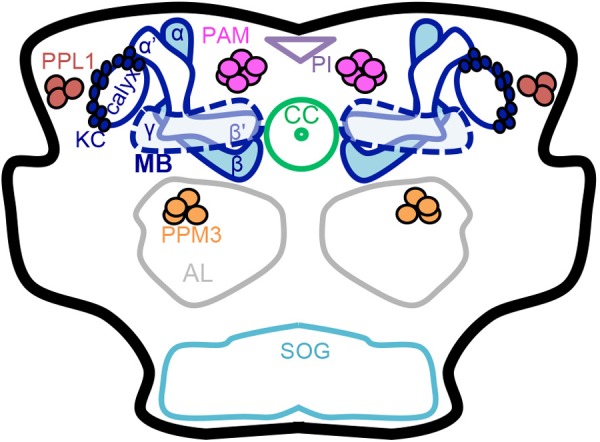
Schematic of the adult *Drosophila* brain. Dopaminergic neurons (PAM, PPL1 and PPM3 clusters) and the mushroom body have a role in learning, memory, natural reward, and drug effects. Dopaminergic neurons have been thoroughly characterized anatomically into defined clusters that project to specific regions in the mushroom body lobes. Mushroom body output neuron connections with intrinsic mushroom body Kenyon cells and dopaminergic neurons form 15 compartments that appear to be functionally independent (Mao and Davis, 2009 ; Chiang et al., 2011 ; Aso et al., 2014a ; Das et al., 2016 ; Kaun and Rothenfluh, 2017). MB mushroom body (α/β, α′/β′, and γ lobes) (blue), KC Kenyon cells (blue), CC central complex (light green), AL antennal lobe (gray), SOG subesophageal ganglion (teal), PAM protocerebral anterior medial neurons (pink), PPM3 protocerebral posterior medial 3 neurons (orange), PPL1 protocerebral posterior lateral 1 neurons (brown), PI pars intercerebralis (light purple).

The proboscis extension reflex (PER) can be used to measure the palatability of a stimulus. Sugars are detected by gustatory receptor neurons located in the tarsae and mouthparts of *Drosophila* (Wang et al., 2004 ; Amrein and Thorne, 2005). PER response to sugar is partly mediated by Gr5a, a gustatory receptor expressed in specific sensory neurons (Wang et al., 2004). It has been shown that both sweet and nutritious and also sweet but non-nutritious sugars elicit PER responses in flies (Dus et al., 2011 ; Fujita and Tanimura, 2011). A single dopaminergic ventral unpaired medial neuron (TH-VUM), which has projections in the SOG, is sufficient to elicit a PER response to sucrose (**Figure 1**) (Marella et al., 2012). The DA receptor 2 (*Dop2R*) was required for DA-induced PER (Marella et al., 2012). Another neurotransmitter involved in the PER is serotonin (Albin et al., 2015). Activating a subset of serotonergic neurons, R50H05, increased PER responses in sated flies, which normally would be low (Albin et al., 2015).

The number of trials to the first PER response is an indication of sucrose responsiveness and has been shown to correlate with the PER habituation, which is the reduction of PER response upon repetition of the stimulus (Çevik and Erden, 2012). Flies with lower sucrose responsiveness habituate faster, and flies with high sucrose responsiveness habituate slower (Scheiner et al., 2014). OA was implicated in PER habituation indirectly, via modulation of sucrose responsiveness (Scheiner et al., 2014). Flies with mutations in the gene for the rate limiting enzyme for OA synthesis, *Tyramine β hydroxylase* (*Tβh*), have unaffected PER habituation rates but decreased response to sucrose, which can be rescued by supplementing OA by feeding or by expressing OA specifically in octopaminergic neurons of the ventro medial cluster of the SOG (Scheiner et al., 2014). This suggests that OA promotes sucrose responsiveness (Scheiner et al., 2014).

#### Voluntary Consumption and Two-Choice Preference

Flies display preference for sugars, but this preference is not solely based on taste. In a two-choice preference assay, flies first chose the sweetest sugar but after 5 min, flies started favoring the nutritious sugar (Dus et al., 2015). The continued ingestion of nutritious sugar induced PER response and activated food processing in the gut (Dus et al., 2015). Flies develop preference for nutritious sugar even in the absence of taste input, but only after a long period of starvation (Dus et al., 2011). After 5 h of starvation, which correlates with decreased hemolymph levels of glucose and trehalose, taste receptor mutant flies preferentially ate agar with sucrose in a two-choice agar plate (Dus et al., 2011). Another study using the two-choice CApillary FEeder (CAFE) assay confirmed that flies choose sugars according to sweetness but that this initial preference shifts toward sugars with higher nutritional value after 12 h, which suggests that this phenomenon is experience dependent (Ja et al., 2007 ; Stafford et al., 2012).

Preference for a nutritious sugar is mediated by Dh44 neurons, which produce and release the Diuretic hormone 44 neuropeptide (the homolog of corticotropin-release hormone in mammals), which activates the Dh44 receptor R1 (Dus et al., 2015). Dh44 neurons mediating preference for nutritious sugars are located in the PI and their neurites project to the dorsal region of the subesophageal zone (the basal region of the supraesophageal ganglion fused to the SOG) and also innervate the gut (Dus et al., 2015 ; Hartenstein et al., 2018). In Dh44 neurons, a nutritious sugar stimulus causes changes in calcium oscillation frequency and duration, suggesting neuropeptide secretion (Thorner et al., 1988 ; Dus et al., 2015). Sugar transport into Dh44 cells and glucose metabolism are necessary to induce calcium oscillations and for nutritious sugar choice (Dus et al., 2015). These results show that nutritious sugars directly activate Dh44 neurons via a sugar-metabolism-dependent pathway resulting in Dh44 neuropeptide secretion, which conveys the signal of nutritious sugars to other regions of the brain.

Dh44 binds to and activates two receptors in *Drosophila* : R1, expressed in the brain and ventral nerve chord, and R2, expressed in the gut (Dus et al., 2015). The Dh44 receptors R1 and R2 are necessary for preference for nutritious sugars (Dus et al., 2015). Dh44 R1 neuron activation elicits PER responses, while the *Dh44 R2* gene is implicated in gut motility (Dus et al., 2015). Dh44 R1 expressing neurons have neurites in the PI and in the dorsal region of the subesophageal zone (Dus et al., 2015). Projections of Dh44 R1 neurons do not contact muscles in the labella and thus Dus et al. (2015) propose that these neurons synapse onto motoneurons in the subesophageal zone, which would in turn elicit PER (Dus et al., 2015).

The cAMP pathway has been implicated in learning, memory, and reward in *Drosophila*, and it was found that the cAMP pathway in neurons also mediates preference for nutritious sugars (Davis and Kiger, 1981 ; Tempel et al., 1983 ; Schwaerzel et al., 2003 ; Stafford et al., 2012). The shift in preference toward nutritious sugars occurred faster in hungry flies (Stafford et al., 2012). The insulin pathway is also involved in nutritious sugar preference (Stafford et al., 2012). Both the insulin receptor (*InR*) in the thoracic ganglion and the insulin-like peptides *dilp2* and *dilp3* in adult MNC mediate preference for nutritious sugars (Ikeya et al., 2002 ; Stafford et al., 2012). The serotonergic pathway has also been implicated in nutritious sugar preference (Albin et al., 2015). Activation of the R50H05 subset of serotonergic neurons in sated flies increased their preference for a nutritious sugar, mimicking the effects of starvation on nutritious sugar preference (Albin et al., 2015).

**FIGURE 2:**
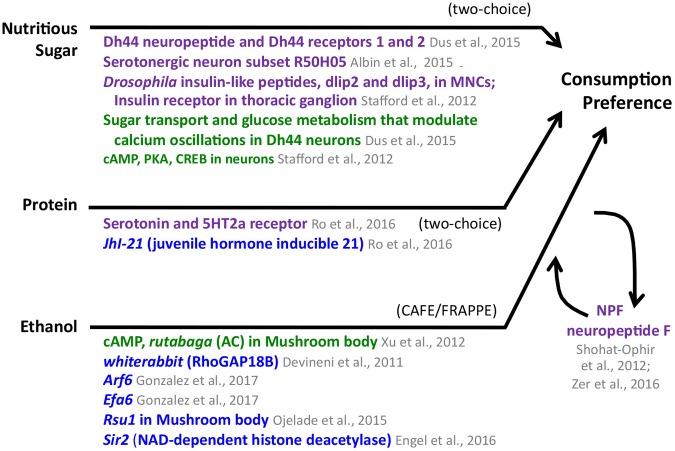
Consumption preference for nutritious sugar, protein, and ethanol in adult *Drosophila*. The appetitive stimulus is shown on the left side, and the behavior, as measured in specific assays, is shown on the right. The compiled genes, signaling pathways, and neural structures mediating the behavior connect the stimulus and the behavior. Consumption preference for nutritious sugar and protein was tested using a two-choice preference assay; ethanol consumption preference was identified using the CAFE/FRAPPE assay. Neuronal pathways (dark purple), cellular pathways (dark green), genes/proteins (royal blue). MNCs median neurosecretory cells.

**Figure 2** summarizes results from the studies about sugar preference above, which suggest that preference for a nutritious sugar is mediated by the cellular signaling cAMP pathway along with the insulin, Dh44 neuropeptide, and a subset of serotonergic neurons. This subset of serotonergic neurons also has a role in the PER for sugars, which are perceived by gustatory receptor neurons.

Hence, sugars are palatable for flies and flies use different neurotransmitter systems to convey hunger signals based on nutrient levels, which are correlated to calcium oscillations. Hunger modulation results in increased PER responses and preference for a nutritious sugar. Next, we examine the evidence supporting sugars as natural rewards for fruit flies and the mechanisms of sugar as reinforcement for short and long-term memories.

### Sugar Reward: Appetitive Olfactory Conditioned Memory

Sugar has been used by multiple investigators as an US in olfactory conditioned learning and memory assays. Quinn et al. (1974) pioneered reward research in *D. melanogaster*, developing a classical conditioning assay in which a neutral odor and an aversive stimulus are paired (Tully and Quinn, 1985). The appetitive version of this assay uses sugar as attractive stimulus, pairing it to an odor (Tempel et al., 1983). This pioneering work identified mutant flies (*rutabaga* and *dunce*) with mutations in the cAMP pathway that had sugar memory defects (Tempel et al., 1983). Later it was shown that this defect could be rescued when wild-type *rutabaga* was expressed specifically in mushroom body Kenyon cells (Schwaerzel et al., 2003). Early studies tested involvement of the cAMP pathway shortly after training (Schwaerzel et al., 2003). It was subsequently shown that the cAMP pathway is also needed in *Drosophila* mushroom body for long-term appetitive memories (Krashes and Waddell, 2008).

The dopa decarboxylase (*Ddc*) gene, whose gene product catalyzes the synthesis of DA and serotonin, was also found to mediate sugar learning (Tempel et al., 1984). Involvement of OA has also been shown, as flies with mutations in *Tβh*, the enzyme that converts tyramine to OA, were impaired in sugar memory performance. This phenotype was rescued by expressing wild-type *Tβh* in mutant flies (Schwaerzel et al., 2003).

Some sugars are perceived as sweet and some are not; some sugars can be metabolized and some cannot (Burke and Waddell, 2011 ; Fujita and Tanimura, 2011). To further probe appetitive learning, researchers investigated whether the sweetness or the nutritional value could both function as reinforcements in appetitive learning and how the brain encodes these two aspects of sugar. Flies can learn the nutritional value of a non-sweet stimulus such as D -sorbitol in an olfactory conditioned memory assay; this learning is dependent on synapsin (*Syn*) (Fujita and Tanimura, 2011). Flies can form short-term memories with nutritious sugars sucrose or fructose and also with arabinose or xylose, which cannot be metabolized by flies (Burke and Waddell, 2011). However, long-term memory formation is much stronger for the nutritious sugars (Burke and Waddell, 2011).

#### Octopamine and Dopamine Mediate Short-Term Memories With Sweet Sugars as Reinforcement

Next, studies identified the neurotransmitter systems that convey sweetness versus those that convey nutritional information. OA signaling is needed for flies to form short-term appetitive memories with sweet taste as reinforcement (Burke et al., 2012). However, OA-dependent memories require DA signaling as well (Burke et al., 2012). Activation of a subcluster of dopaminergic neurons in the PAM cluster is sufficient to induce appetitive olfactory memory in starved flies, showing that DA signaling is downstream of OA-mediated short-term appetitive memory formation (Burke et al., 2012 ; Liu et al., 2012). Neurons in this PAM subcluster have dendrites in the anterior medial protocerebrum and presynaptic terminals in the tip of the mushroom body β′ and γ lobes. GFP reconstituted across synaptic partners (GRASP) analysis suggests that octopaminergic neurons make synapses with neurons in this PAM dopaminergic subcluster (Burke et al., 2012). The subgroup of PAM dopaminergic neurons that mediate OA-dependent olfactory memories express the Ca^2+^-coupled α-adrenergic -like OA (OAMB) receptor, which is necessary for OA-dependent memories (Burke et al., 2012). A subset of OAMB OA receptor neurons within the PAM cluster project to the β′_2 am_ and γ_4_ regions of the mushroom body and convey the short-term reinforcing effect of sweet taste (Huetteroth et al., 2015).

Octopamine also mediates olfactory memories via activation of the octopaminergic receptor, Octβ2R expressed in MB-MP1 dopaminergic neurons, which are part of the dopaminergic PPL1 cluster and innervate the mushroom body heel (γ_1_, α/β peduncle) (Krashes et al., 2009 ; Aso et al., 2010 ; Burke et al., 2012). Burke et al. (2012) proposed a model in which OA mediates appetitive reinforcement via OAMB signaling by modulating the activity of positive PAM dopaminergic neurons and Octβ2R signaling by modulating the activity of negative PPL1 MB-MP1 dopaminergic neurons (Burke et al., 2012).

The *Drosophila* DA 1-like receptor 1 (DopR1) expressed in mushroom body intrinsic neurons (Kenyon cells) is required for OA-mediated appetitive short-term memory (Kim et al., 2007 ; Burke et al., 2012 ; Liu et al., 2012). It has not been tested whether DopR1 is needed in the specific mushroom body compartments that mediate sweet taste short-term memories.

#### Dopamine Mediates Long-Term Memories With Nutritional Sugars as Reinforcements

Octopamine signaling and PAM dopaminergic neurons expressing the OAMB receptor are not required for nutritional value reinforced memories (Burke et al., 2012). A different subset of PAM neurons mediates nutritional value reinforced olfactory memory formation (Burke et al., 2012). PAM dopaminergic neurons that project to the γ_5b_ region of the mushroom body convey the long-term reinforcing effect of nutritional value (Huetteroth et al., 2015). Activation of the dopaminergic neurons innervating the β_1_, β_2_, and adjacent α_1_ regions of the mushroom body is sufficient for long-term memory (Huetteroth et al., 2015 ; Yamagata et al., 2015). Among these sets of PAM neurons, blocking PAM-α_1_ neurons impaired long-term memory formation with a non-nutritious sugar supplemented by a non-sweet nutritious sugar without affecting short-term memory formation, and selective activation of these neurons in hungry flies induced long-term appetitive memory (Yamagata et al., 2015). Hence, PAM-α_1_ neurons are necessary and sufficient for long-term memory formation (Yamagata et al., 2015). PAM-α_1_ neurons receive input from glutamatergic MBON-α_1_ neurons, a specific type of mushroom body output neuron with dendrites in the α_1_ mushroom body compartment (Ichinose et al., 2015). Moreover, PAM-α_1_ neurons and MBON-α_1_ neurons are required for acquisition and consolidation of long-term appetitive memories (Ichinose et al., 2015).

In addition to their role in short-term memory formation, PPL1 MB-MP1 dopaminergic neurons are also necessary and sufficient to convey the nutritional value to the mushroom body (Musso et al., 2015). PPL1 MB-MP1 neuron activity is needed for the establishment of long-term memory after training but not during training (Musso et al., 2015).

The *Drosophila* DA 1-like receptor 2 (DopR2) expressed in mushroom body neurons mediates appetitive long-term memories; this receptor seems to be activated by PPL1 MB-MP1 dopaminergic neurons signaling (Musso et al., 2015).

#### Hunger Modulation of Sugar Memories

Dopaminergic PPL1 MB-MP1 neurons express the NPF receptor 1 (NPFR1); NPF is the *Drosophila* homolog of mammalian NPY. Dopaminergic PPL1 MB-MP1 neurons are inhibited by NPF in hungry flies, allowing for the retrieval of appetitive memories (Krashes et al., 2009). PPL1 MB-MP1 neurons seem to function as a gate at the mushroom body, providing tonic inhibition when flies are fed and relieving this inhibition when they in turn become inhibited by NPF during food deprivation (Krashes et al., 2009). NPF stimulation increases appetitive memory performance in fed flies, mimicking performance of hungry flies (Krashes et al., 2009). Hence, starvation modulates appetitive olfactory memory formation centrally via NPF signaling at the PPL1 MB-MP1 dopaminergic neurons.

PPL1 MB-MP1 neurons have spontaneous calcium oscillations that change according to hunger state (Plaçais et al., 2012 ; Plaçais and Preat, 2013). These oscillations increase in frequency and quality factor 30 min after training with a nutritious sugar compared to training with a non-nutritious sugar (Musso et al., 2015). This delayed calcium trace in PPL1 MB-MP1 neurons correlates with the nutritional value of the sugar reward and with appetitive long-term memory formation (Musso et al., 2015). More recently, it has been shown that a subset of serotonergic neurons encodes the hunger signal. Activating these neurons results in fed flies eating as if they were starved (Albin et al., 2015).

Musso et al. (2015) proposed a two-step mechanism for appetitive memory formation: (1) integration of olfactory and gustatory sensory information and (2) post-ingestion energetic value (Musso et al., 2015). The nutritional value of food is the critical signal for generating long-term memory (Musso et al., 2015). Flies develop long-term memories when given a non-nutritious sugar only when fed a nutritious sugar immediately after training to mimic a post-ingestion signal (Musso et al., 2015). Long-term memory formation is impaired when intestinal glucose transport is blocked, which lowers glucose levels in the hemolymph (Musso et al., 2015). Sugar levels in hemolymph after sugar ingestion may represent their nutritional value (Yamagata et al., 2015). Fructose is sensed by the Gr43a receptor in the brain. Blocking Gr43a-expressing neurons during appetitive reward training impaired long-term memory formation while sparing short-term memory (Yamagata et al., 2015). Gr43a expressing neurons and their neuronal projections locate to the lateral protocerebrum in the same region where dendrites from PAM neurons that mediate long-term memories are located (Yamagata et al., 2015).

**FIGURE 3:**
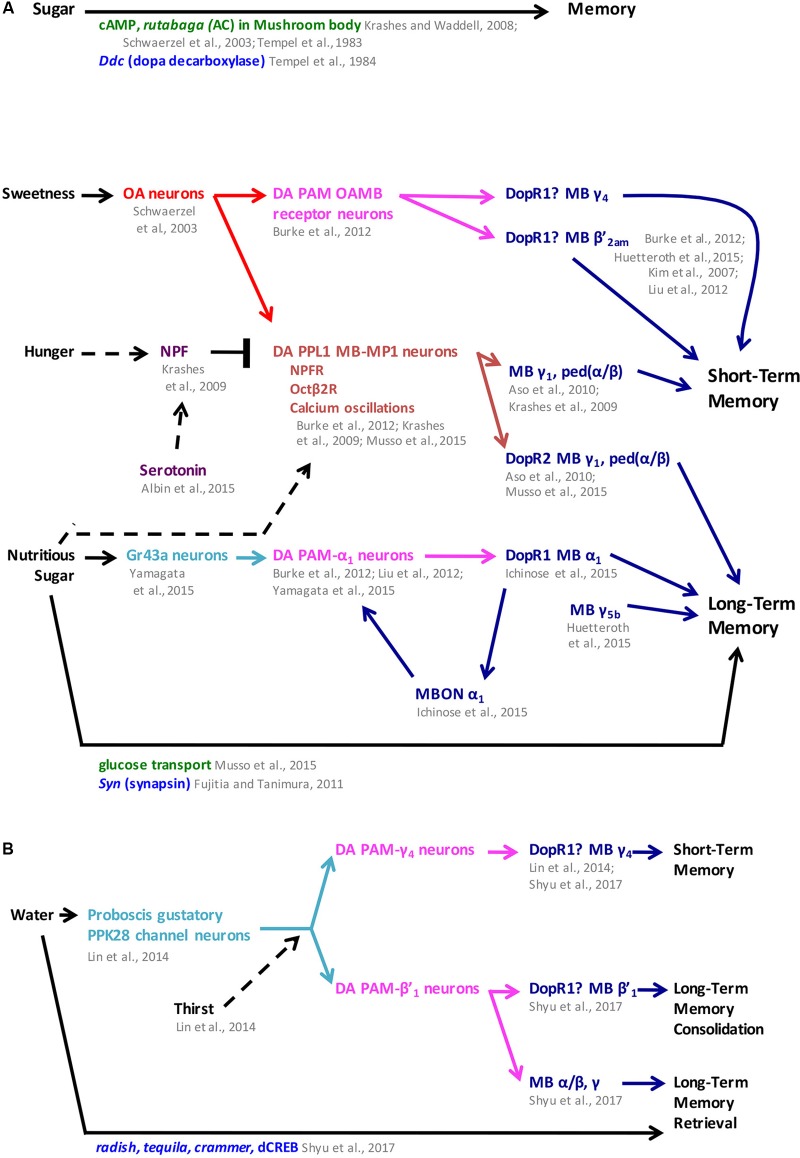
Sugar and water memory follow parallel pathways in *Drosophila*. **(A)** Sweetness and nutritional value reinforce short- and long-term memory formation, respectively, through dopaminergic-mushroom body circuitry with hunger modulating both short- and long-term memory. **(B)** Water memory is mediated by dopaminergic PAM clusters through the mushroom body and is modulated by thirst. In some cases, mushroom body compartments were identified independently from the dopaminergic receptor function. In the figure, question marks next to dopaminergic receptors indicate when their function has been localized to the mushroom body but has not been narrowed down to a specific compartment. MB mushroom body (α/β, α′/β′, and γ lobes) (blue), MBON mushroom body output neurons (blue), OA octopaminergic (red), gustatory neurons (teal), PAM protocerebral anterior medial (pink), PPL1 protocerebral posterior lateral 1 (brown), neuronal pathways (dark purple), cellular pathways (dark green), genes/proteins (royal blue). NPF neuropeptide F; ped (α/β): peduncle of α/β.

**Figure 3** summarizes the findings showcased above, which demonstrate that sugars are natural rewards with the ability to serve as reinforcements for both short and long-term memories in *D. melanogaster*. The mechanisms underlying sugar reward show that parallel pathways for short versus long-term memory exist in the fly brain and each pathway involves different sets of neurotransmitters systems: OA and DA for sweet taste short-term memories, and DA for nutritious value long-term memories. These parallel circuits and the role of DA as a central neurotransmitter in reward memory formation reveals that *Drosophila* reward circuits are surprisingly more similar to mammals than previously thought. This further validates fruit flies as a valuable model organism to help elucidate the organizing principles of the reward circuits to complement research in mammalian systems.

Sugar reward in fruit flies has been studied the most. However, research to determine palatability and preference of other natural food stimuli in the context of reward has begun to reveal interesting similarities and differences to sugar reward mechanisms.

### Protein and Fatty Acid Palatability and Preference

Medium-chain fatty acids elicit PER responses at a significantly higher rate than water (Masek and Keene, 2013). Medium-chain fatty acids are sensed by peripheral sugar-sensing sensory receptor neurons that express the Gr64f receptor. Silencing these neurons abolishes not only the PER response to sugar, but also the PER response to the medium-chain fatty acids (Masek and Keene, 2013). The Phospholipase C (PLC) homolog “no receptor potential A” (*norpA*) is required in Gr64f neurons for PER responses to fatty acids. *norpA* mutants have significantly lower PER responses to fatty acids, while the PER responses to sugar are unaffected (Masek and Keene, 2013). Neurons expressing the ionotropic receptor 56d (IR56d) respond to short and medium-chain fatty acids; *norpA* is also required in these neurons for fatty acid PER responses (Tauber et al., 2017). A subpopulation of neurons that co-express Gr64F and IR56d mediates fatty acid taste and PER responses (Tauber et al., 2017). Even though these neurons respond to both sucrose and fatty acids, flies can distinguish between these two stimuli and form independent memories for sugar and fatty acids in an aversive memory test (Tauber et al., 2017).

In addition to eliciting PER, flies also prefer medium-chain fatty acids over water or low concentrations of sucrose (<1 mM) in the two-choice CAFE assay (Masek and Keene, 2013). Flies can also develop preference for protein. Starved flies developed preference for sugar food with added protein over sugar alone, while fed flies preferred sugar-only food (Ro et al., 2016). This protein preference is mediated by serotonin signaling acting through the 5HT2a receptor (Ro et al., 2016). Serotonergic signaling is needed during starvation to form protein preference but is not necessary during food-choice (Ro et al., 2016). Activation of serotonergic neurons results in protein preference in fed-flies, which suggests that serotonin increases the value of protein-food and that this value changes according to energy state (Ro et al., 2016). Protein preference is also mediated by the juvenile hormone inducible 21 (*JhI-21*) gene, a homolog of SLC7A5 (a mammalian leucine transporter), and seems to act upstream of serotonin signaling (Ro et al., 2016).

A summary of the results from the studies above is found in **Figure 2**. Flies find fatty acids palatable, based on their ability to elicit PER responses, and there seems to be some overlap with sugar palatability. It would be interesting to determine whether proteins also elicit PER responses. Flies show preference for both fatty acids and protein. Protein preference is mediated by *JhI-21* and serotonergic signaling. The mechanism mediating fatty acid preference is not currently know. It would be interesting to determine whether fatty acids or proteins can act as reinforcements in either short or long-term memory and whether these memories are encoded by additional, not yet identified parallel pathways to those of sugar memories. The study of another natural stimulus, water, suggests there are indeed additional parallel pathways for conveying different natural stimuli.

### Water Reward

Water is rewarding for thirsty flies, as tested in a 3-min water-mediated learning assay or a 30-min water short-term memory assay (Lin et al., 2014). In flies, Pickpocket 28 (PPK28), an osmosensitive channel expressed in gustatory neurons in the proboscis, detects water (Cameron et al., 2010). Flies avoid water when not thirsty, but display approach behavior after water deprivation (Lin et al., 2014). Pairing water with a neutral odor is an effective reinforcement in an olfactory appetitive learning assay and is conveyed by a specific subpopulation of dopaminergic neurons separate from those involved in sugar reward (Lin et al., 2014). Pickpocket 28 mediates water reinforcement, as *ppk28* mutants are deficient in water-dependent learning but are able to detect water and other smells (Lin et al., 2014). A subset of PAM cluster dopaminergic neurons with projections to the γ_4_ region of the mushroom body was required for water learning acquisition (Lin et al., 2014). The DopR1 receptor was required in γ lobe mushroom body neurons for water learning, while OA was not required (Lin et al., 2014). Naï ve water-seeking behavior is mediated by a different pathway than water-learning behavior. PAM neurons innervating the β′_2_ region of the mushroom body lobe mediated naï ve water seeking, but the DopR1 receptor was not involved (Lin et al., 2014).

Another study distinguished short- versus long-term water memories, and identified additional dopaminergic clusters that mediate these memories (Shyu et al., 2017). PAM-γ_4_ neurons mediate short-term water memory in thirsty flies (Lin et al., 2014 ; Shyu et al., 2017). Water reward also produces a protein synthesis-dependent long-term memory when tested 24 h after conditioning. Long-term water memory is disrupted by cycloheximide, and is also negatively affected in *radish*, *crammer*, *tequila*, and *dCREB* mutants. Long-term water memory is mediated by PAM dopaminergic neurons that innervate the β′_1_ region of the mushroom body lobes (Shyu et al., 2017). The DopR1 DA receptor is required in α′/β′ neurons for long-term water memory (Shyu et al., 2017). Different subsets of mushroom body neurons are required for consolidation and retrieval of long-term water memories. Output from α′/β′ is needed for consolidation, while output from γ and α/β neurons is needed for memory retrieval (Shyu et al., 2017).

**Figure 3** summarizes the findings about water reward and highlights the similarities and differences in the pathways that mediate sugar memories versus those that mediate water memories. Next, we switch focus to what is currently known about drug reward in *D. melanogaster*. Ethanol has been studied the most and has been shown to be rewarding for fruit flies. Comparison of genes, neurotransmitters and neural circuits that are involved in locomotor effects of ethanol against those for ethanol reward reveal overlap, suggesting shared mechanisms. With this insight, we include data from locomotor assays for additional drugs of interest. Genes, molecular pathways, and neural circuits underlying locomotor drug effects may provide hints for additional mechanisms for drug reward to be investigated in the future.

## Drug Reward

Reward systems require the integration of sensory information and the formation of memory to assign beneficial or harmful associations to the stimuli and result in motivated behavior. There are three main theories of addiction. The incentive sensitization theory of addiction postulates that repetitive exposure to drugs of abuse persistently modifies the neurons and neural circuits that mediate incentive salience attributed to the drug stimulus and also drug-associated cues to the point of reaching a pathological level of ‘wanting’ for the drug (Robinson and Berridge, 2008). This theory of addiction focuses heavily on ‘wanting’ and its neural correlate of mesolimbic DA sensitization, which is most common after repeated, spaced apart, high dose exposure to drugs (Berridge and Robinson, 2016). A second theory of addiction has developed around the concept of allostasis and opponent-process theory, including changes in neurotransmitter systems, neural circuits, and stress systems that result in an alternative homeostatic condition in response to drugs of abuse (Koob and Le Moal, 2008 ; Wise and Koob, 2014). This leads to a ‘chronic elevation of reward set point’ (Koob and Le Moal, 2008 ; Wise and Koob, 2014). Lastly, the third theory of addiction attributes the shift from voluntary drug taking to compulsive drug abuse to alterations in neurocircuitry involving habit systems and the development of ‘habit-based learning’ (Everitt et al., 2008 ; Everitt and Robbins, 2016). These theories continue to evolve as we gain insight into the mechanisms of both natural and drug reward.

Drug reward research in *D. melanogaster* has focused on identifying genes and neural circuits underlying the reinforcing properties of drugs. In the next section of this review, we discuss palatability, preference, and rewarding properties of ethanol. We delve into the genetic and neural mechanisms of ethanol’s locomotor effects, which include changes in neurotransmitter systems and neural circuits. Lastly, we compiled data on mechanisms mediating the locomotor effects of cocaine, amphetamine, methamphetamine and nicotine.

### Ethanol: Palatability and Preference

#### Palatability: Proboscis Extension Reflex

Studies have shown that ethanol is not an appetitive tastant for flies upon initial exposure. In one study, ethanol concentrations ranging from 0.1 to 40% failed to elicit PER responses (Devineni and Heberlein, 2009). When these concentrations were mixed with 100 mM sugar, which elicits reliable PER responses, there was an ethanol-concentration-dependent decrease in PER response frequency (Devineni and Heberlein, 2009). These results were replicated by Xu et al. (2012), who showed that ethanol preference did not significantly decrease PER responses for ethanol-laced sucrose food at low ethanol concentrations (Xu et al., 2012).

However, Devineni and Heberlein (2009) found that flies develop preference for ethanol-laced food over time, with flies exhibiting a mild preference for ethanol after a single day of consumption and increasing preference over the next 4 days (Devineni and Heberlein, 2009). Even though ethanol is not palatable to naï ve flies, flies prefer olfactory traps with an ethanol smell, showing that ethanol smell is attractive to flies (Devineni and Heberlein, 2009 ; Schneider et al., 2012). It would be interesting to test PER in flies that have developed preference for ethanol.

#### Voluntary Consumption and Two-Choice Preference

Devineni and Heberlein (2009) modified the capillary feeder (CAFE) assay by Ja et al. (2007) to quantify voluntary ethanol consumption over time in chambers that included a choice between food laced with ethanol and food without the ethanol (Ja et al., 2007 ; Devineni and Heberlein, 2009). A comparison between the amount of ethanol food versus non-ethanol food consumed over time was then used to calculate a preference index. Results from this assay showed that flies develop a dose-dependent preference for food containing ethanol. Pohl et al. (2012) also showed that flies prefer ethanol-containing food (Pohl et al., 2012). Flies increase their ethanol consumption over time, are willing to overcome an aversive stimulus of quinine to consume ethanol food, and will go back to ingesting large amounts of ethanol following a deprivation period (Devineni and Heberlein, 2009). Ethanol preference in the CAFE assay is mediated by the cAMP pathway in the mushroom body. The adenylyl cyclase gene, *rutabaga*, is required in mushroom body neurons for flies to develop ethanol preference (Xu et al., 2012). Further investigation will be required to determine which specific mushroom body neurons mediate ethanol preference.

Ethanol preference in the CAFE assay was not based on nutritional value, as flies are not able to efficiently utilize ethanol calories for survival (Xu et al., 2012). The FRAPPE, a novel high-throughput ethanol consumption preference assay that measures the consumption of individual flies, further showed that ethanol preference in *Drosophila* is not driven by calories (Peru y Colón de Portugal et al., 2014). This study demonstrated that ethanol preference in fruit flies is an experience-dependent process in which ethanol is mildly aversive to naï ve flies. However, flies develop long-lasting preference for ethanol food after 20 min of ethanol vapor pre-exposure (Peru y Colón de Portugal et al., 2014). Flies also developed ethanol preference when the pre-exposure was achieved by pre-feeding flies with ethanol-laced food both in a no-choice and in a two-choice configuration in the CAFE. This result shows that different routes of ethanol pre-exposure all lead to ethanol preference (Peru y Colón de Portugal et al., 2014).

A follow up study by Devineni et al. (2011) identified additional genes that regulate voluntary ethanol consumption, including *whiterabbit*, which codes for RhoGAP18B, a GTP-ase activating protein of the Rho family (Devineni et al., 2011). Flies with *whiterabbit* mutations had decreased voluntary ethanol consumption in the two-choice CAFE assay (Devineni et al., 2011). Other genes shown to act in the same pathway as RhoGAP18B also have ethanol consumption phenotypes. Unlike wild-type flies that require ethanol pre-exposure to develop preference for ethanol, naï ve *Arf6* and *Efa6* mutant flies display a high and unchanging preference for ethanol food (Gonzalez et al., 2017). *Rsu1* mutants also have naï ve preference for ethanol and acquire ethanol preference over time. A targeted decrease in *Rsu1* in the mushroom body resulted in flies with no naï ve preference for ethanol or gradual ethanol preference, which showed that *Rsu1* in the mushroom body mediates gradual ethanol preference, while *Rsu1* acts in neurons outside the mushroom body to mediate naï ve preference (Ojelade et al., 2015). Another gene, *Sir2*, also mediates ethanol preference and encodes for NAD-dependent histone deacetylase sirtuin-2 (Engel et al., 2016). *Sir2* mutant flies have high naï ve preference for ethanol food but fail to develop ethanol preference after ethanol pre-exposure (Engel et al., 2016).

Ethanol preference can be modified by social experience, specifically sexual experience (Shohat-Ophir et al., 2012). Sexually rejected males have higher ethanol consumption and ethanol preference than mated males (Shohat-Ophir et al., 2012). Mating status was correlated with levels of NPF (Shohat-Ophir et al., 2012). NPF transcript and protein levels were higher in mated males compared to rejected males (Shohat-Ophir et al., 2012). NPF pathway activity mediated ethanol preference, increasing ethanol preference when it was downregulated and decreasing ethanol preference when it was artificially activated (Shohat-Ophir et al., 2012). Notably, activation of the NPF pathway was found to be rewarding for flies in a conditioned odor preference assay (Shohat-Ophir et al., 2012). In addition, artificial activation of the NPF pathway abolished the preference for ethanol (Shohat-Ophir et al., 2012). It was also shown that the ethanol exposure regime that was rewarding for flies increased NPF levels (Shohat-Ophir et al., 2012). This study suggests a homeostatic model of reward in which the NPF pathway signals reward level status in *Drosophila*. This means that experiences that lower NPF signaling promote reward-seeking behaviors, while experiences that increase NPF signaling decrease reward-seeking behaviors (Shohat-Ophir et al., 2012 ; Devineni and Heberlein, 2013). These results have been replicated in a methods paper that details this novel experimental design to study reward in the fly (Zer et al., 2016). This experimental design has two components: the first consists of exposing the flies to either rewarding or non-rewarding experiences and the second consists of determining their voluntary ethanol consumption as a measure of motivation to seek a drug reward (Zer et al., 2016). This assay can be used to study how experience modulates drug reward and to identify novel genes and neural circuits that mediate reward (Zer et al., 2016).

**Figure 2** summarizes the studies on palatability and preference for ethanol. The experience-dependent and delayed preference for ethanol described above is reminiscent of how preference for a nutritious sugar develops. One similarity is the role of the cAMP pathway as a mediator of both sugar and ethanol preference. It would be interesting to test if ethanol elicits calcium oscillations, as sugar does. There are also differences, for example the involvement of RhoGAP18 and the NAD-dependent histone deacetylase sirtuin-2 in ethanol preference.

Neuropeptide F is also a common factor between sugar and ethanol. NPF has been shown to be involved in hunger modulation of sugar memories by inhibiting specific dopaminergic neurons and allowing retrieval of sugar memories in hungry flies (**Figure 3**). Research on the role of NPF in ethanol preference has shown a negative correlation between levels of this neuropeptide and ethanol preference, either promoting ethanol consumption when NPF levels are low or decreasing ethanol consumption when NPF levels are high. A similar scenario for sugar would be that high levels of NPF correlate with hunger, which increases appetitive olfactory memory performance, a measure of increased sugar reward; low levels of NPF correlate with the sated state, which decreases appetitive memory performance and sugar reward. More is known about how NPF levels are modulated by hunger. It will be interesting to determine if similar mechanisms affect either sweet or nutritious sugar preference.

#### Oviposition Preference for an Ethanol Substrate

Flies also display preference for ethanol as a substrate for oviposition. It has been shown that flies prefer a substrate with 5% ethanol on a two-choice oviposition preference assay (Azanchi et al., 2013). Flies are attracted to acetic acid or the bitter compound lobeline for oviposition, while displaying positional aversion for these substrates, and the mushroom body was implicated in these behaviors (Joseph et al., 2009 ; Joseph and Heberlein, 2012). Flies did not show positional aversion or attraction to ethanol at the concentrations that elicited oviposition preference (Azanchi et al., 2013). Dopaminergic neurons of the PAM and the PPM3 clusters promote oviposition preference for ethanol, while PPL1 MB-MP1 neurons in the PPL1 cluster inhibit oviposition preference for ethanol (Azanchi et al., 2013). Both PAM and PPL1 dopaminergic neurons innervate the mushroom body, while PPM3 neurons innervate the ellipsoid body of the central complex (Mao and Davis, 2009 ; Kong et al., 2010b ; Aso et al., 2012). The α′/β′ mushroom body neurons mediated oviposition preference, as did the ring R2 neurons of the ellipsoid body (Azanchi et al., 2013). The role of dopaminergic receptors in the mushroom and ellipsoid bodies was also tested. It was shown that decreasing DopR2 in the mushroom body increased oviposition preference, while decreasing either DopR1 or DopR2 in the ellipsoid body each had the effect of increasing oviposition (Azanchi et al., 2013). A model was proposed in which the PAM and PPM3 neurons signal an appetitive stimulus and promote oviposition preference, while the PPL1 MB-MP1 neurons signal an aversive stimulus and suppress oviposition preference (Azanchi et al., 2013).

**FIGURE 4:**
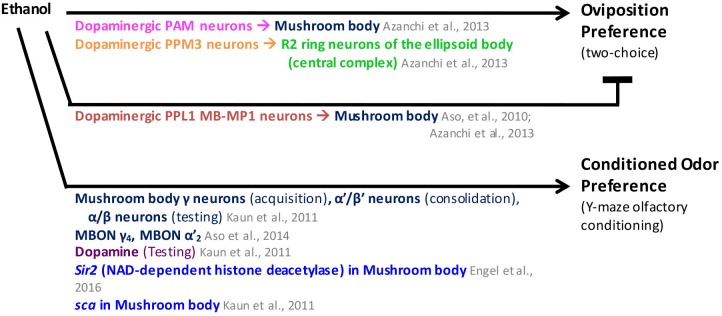
Adult *Drosophila* choose an ethanol-associated stimulus in oviposition and conditioned odor preference assays. MB mushroom body (α/β, α′/β′, and γ lobes; blue), MBON mushroom body output neurons (blue), CC central complex (light green), PAM protocerebral anterior medial neurons (pink), PPL1 protocerebral posterior lateral 1 neurons (brown), neuronal pathways (dark purple), cellular pathways (dark green), genes/proteins (royal blue).

**Figure 4** summarizes the findings described above. Even though oviposition preference for ethanol at first glance may seem a very different assay to sugar preference or sugar reward, the apparent similarities in the neural circuits warrant further consideration about what this assay may be able to tell us about reward. Indeed, there are also similarities in the mechanisms underlying oviposition preference for ethanol and conditioned odor preference for ethanol, as shown in the next section.

### Ethanol Reward: Conditioned Odor Preference

The conditioned odor preference assay for ethanol reward developed by Kaun et al. (2011) is the most sophisticated drug reward assay for *Drosophila*, and was designed with the specific goal of establishing a model of drug reward using *D. melanogaster* (Kaun et al., 2011). In this assay, neutral odors are paired with a moderately intoxicating dose of ethanol during training. During testing, each odor is streamed from opposite ends of a Y-maze. Flies are placed in the bottom of the Y-maze and given 2 min to climb up the maze, either toward the arm where the odor associated with the ethanol is being streamed or to the arm with an unpaired odor. The number of flies in each side of the Y-maze is counted and a preference index is calculated. Using this assay, Kaun et al. (2011) demonstrated that flies develop conditioned odor preference for moderate concentrations of ethanol that elicit locomotor hyperactivity. Flies showed aversion to ethanol when tested 30 min after training but exhibited conditioned odor preference for ethanol 24 h after training, with preference first detected 12 h after training (Kaun et al., 2011). Conditioned preference for ethanol is dose-dependent and does not occur at lower doses that do not elicit behavioral effects in fruit flies, or at high doses that elicit sedation (Kaun et al., 2011). These results show that ethanol reward is long-lasting (Kaun et al., 2011). Interestingly, flies will overcome an electric shock to reach the Y-maze arm containing the odor associated with ethanol exposure (Kaun et al., 2011).

Dopamine was shown to be required for conditioned odor preference memory expression during preference testing but not during training or memory consolidation (Kaun et al., 2011). Activity in mushroom body neurons was needed for conditioned odor preference in sequence, such that γ neurons were needed during acquisition, α′/β′ neurons during consolidation, and α/β neurons during testing (Kaun et al., 2011). Given that dopaminergic neurons and α/β neurons in the MB were both needed during expression of the ethanol memory, it was proposed that ethanol reward memory is mediated by dopaminergic neurons that innervate the α/β neurons (Kaun et al., 2011). The mushroom body output neurons MBON-γ_4_ and MBON-α′_2_ were involved in conditioned odor preference for ethanol 24 h after training (Aso et al., 2014b). A genetic screen of a subset of mutant strains with GAL-4 reporter expression in the mushroom body identified a strain with persistent aversion that had a mutation in *scabrous* (*sca*), a fibrinogen-related peptide that functions via the Notch pathway and was found to be expressed in α/β and γ mushroom body neurons among other regions (Kaun et al., 2011). Another study showed that *Sir2* mutants had reduced conditioned odor preference for ethanol, suggesting that ethanol is not rewarding for *Sir2* mutants (Engel et al., 2016). *Sir2* appears to be required in mushroom body neurons for ethanol reward, as flies with reduced *Sir2* expression in the mushroom body did not display conditioned odor preference for ethanol (Engel et al., 2016).

A summary of the results from the studies above can be found in **Figure 4**. It is still unknown which dopaminergic clusters convey ethanol memories. However, the similarity between the neurons and neurotransmitter systems involved in oviposition preference for ethanol and conditioned odor preference for ethanol suggests that both are mediated through the same neural circuits (Kaun and Rothenfluh, 2017). In this proposed pathway, ethanol is a stimulus with dual properties: aversion and attraction. Appetitive reinforcement from ethanol exposure would be conveyed by activation of the dopaminergic neurons of the PAM cluster, while aversive reinforcement would be conveyed by activation of the dopaminergic neurons of the PPL1 cluster, (Kaun and Rothenfluh, 2017).

The experiments described above show that ethanol is rewarding to flies and also display preference for ethanol. Studies of ethanol preference demonstrated that this preference can be modulated, identifying NPF as a key modulator. NPF is also a modulator in sugar reward. Future research could determine whether NPF plays a role in modulation of ethanol reward.

In the next section, we move from the traditional assays used to study drug reward to measuring acute drug effects. These assays have identified additional mechanisms of drug action. Some of these mechanisms may provide new insights into genes and molecules that have not yet been implicated in ethanol and natural reward.

### Ethanol Locomotor Effects

Ethanol exposure elicits different locomotor effects, including hyperactivity and loss of postural control. However, flies develop tolerance to these effects when re-exposed to ethanol (Kaun et al., 2012 ; Devineni and Heberlein, 2013). Using these behaviors as a marker for ethanol sensitivity, many genes, molecular pathways and neural structures have been identified as mediators for ethanol’s effects (Kaun et al., 2012 ; Devineni and Heberlein, 2013).

#### Ethanol Hyperactivity

Ethanol exposure can increase locomotion in fruit flies (Wolf et al., 2002). Ethanol hyperactivity is modulated by hunger, with starvation increasing ethanol hyperactivity (Kliethermes, 2013). Interestingly, feeding flies just before exposure to ethanol with standard food or sucrose (but not yeast) blocked this effect of food deprivation (Kliethermes, 2013).

The dopaminergic pathway, specifically, a subset of dopaminergic neurons in the PPM3 cluster, mediates ethanol-induced hyperactivity (Bainton et al., 2000 ; Kong et al., 2010b). These neurons project to the ellipsoid body region of the central complex, known for its role in motor control. Moreover, specific neurons within the ellipsoid body, the ring neurons (R) R2/R4, have been implicated in ethanol-hyperactivity. These neurons express *DopR1*, which is required for ethanol-induced hyperactivity (Kong et al., 2010b). Alcohol dehydrogenase (*Adh*) and the cAMP pathway have also been shown to play a role in ethanol hyperactivity (Wolf et al., 2002). The *whiterabbit* gene, specifically the isoform RhoGAP18B-RA, promotes ethanol hyperactivity (Rothenfluh et al., 2006).

The *tao* gene, which encodes a serine-threonine kinase in the Mst/Ste20 family, has a role in adult nervous system development including mushroom body development (King et al., 2011). α/β mushroom body neurons and Tao through Par-1 mediate ethanol hyperactivity (King et al., 2011). *tao* mutants showed an increase in Tau phosphorylation, a microtubule stabilizing protein that is normally phosphorylated by Par-1. This suggests that *tao* exerts its effect on ethanol hyperactivity through a pathway that controls microtubule dynamics during development (King et al., 2011).

The epidermal growth factor receptor (EGFR) and the fibroblast growth factor receptor (FGFR) pathways have also been shown to modulate ethanol hyperactivity in opposing ways, suppressing and promoting ethanol hyperactivity, respectively (King et al., 2014). EGFR signaling, JNK signaling, and *tao* have been shown to act together in mushroom body development, which is a likely mechanism underlying ethanol hyperactivity (King et al., 2011, 2014).

**FIGURE 5:**
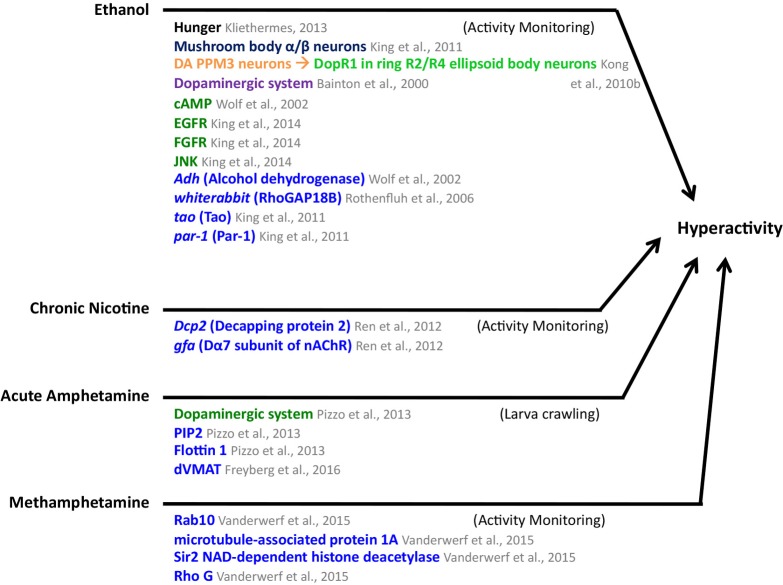
Mediators of ethanol, chronic nicotine, acute amphetamine, and methamphetamine-induced hyperactivity in *Drosophila*. MB mushroom body (α/β, α′/β′, and γ lobes; blue), central complex (light green), PPM3 protocerebral posterior medial 3 neurons (orange), neuronal pathways (dark purple), cellular pathways (dark green), genes/proteins (royal blue). DA, dopaminergic.

**Figure 5** summarizes the findings described above. Similarities between the ethanol hyperactivity and natural reward include the modulation by hunger, the involvement of the dopaminergic and the cAMP pathways.

#### Ethanol Sedation

If flies are exposed to ethanol continuously, the hyperactivity phase is followed by a loss of postural control, and the flies will eventually become sedated. Early work implicated the cAMP pathway in ethanol sensitivity (Moore et al., 1998). The *whiterabbit* gene product, isoform RhoGAP18B-RC, plays a role in ethanol sedation and was shown to function in adult flies through Rho1 and Rac1, which are small GTP-ases (Rothenfluh et al., 2006). A follow up study showed that RhoGAP18B-RC acts together with Rac1, the small GTPase Arf6, and *Drosophila* Arfaptin (Arfip) in adult neurons to regulate ethanol sedation (Peru y Colón de Portugal et al., 2012). Arfip interacts with GTP-bound Arf6 and GTP-bound Rac1, while Arf6 acts downstream of RhoGAP18B, Arfip, and Rac1 to mediate normal ethanol sedation (Peru y Colón de Portugal et al., 2012). Different RhoGAP18B isoforms act via specific Rho-family GTPases, which in turn regulate cofilin activity, an actin depolymerizing protein (Ojelade et al., 2015). Cofilin mutants had decreased sensitivity to ethanol sedation, and functioned downstream of RhoGAP18B-PC and –PD isoforms; these isoforms inhibited Rac1 and in turn regulated cofilin activity, leading to differences in actin dynamics (Ojelade et al., 2015).

The insulin pathway has been previously implicated in ethanol sedation and in mediating the effects of developmental ethanol exposure (Corl et al., 2005 ; McClure et al., 2011). The Insulin receptor (*InR)* is upstream of Arf6, which acts upstream of the p70 S6 kinase (S6k) to modulate ethanol sedation (Acevedo et al., 2015). Completing this ethanol sedation pathway, it was found that integrin signaling is upstream of Rac1 and that Ras suppressor 1 (Rsu1) inhibits Rac1 (Ojelade et al., 2015). A new study added Efa6 to the pathway, which is a guanine exchange factor for *Drosophila* Arf6 (Gonzalez et al., 2017). Like *Arf6* mutants, *Efa6* mutant flies have increased sensitivity to ethanol sedation and it was shown that *Efa6* acts upstream of *Arf6* and normally functions to activate Arf6. Together, *Efa6* and *Arf6* modulate ethanol sensitivity (Gonzalez et al., 2017).

Ethanol sensitivity is also regulated by *dLmo* genes, which are members of the LIM-homodomain transcription factor family that functions in fly circadian pacemaker neurons that express the pigment dispersing factor neuropeptide (Tsai et al., 2004 ; Lasek et al., 2011). The clock gene period (*per*) also modulates ethanol sedation (De Nobrega and Lyons, 2016 ; Liao et al., 2016). The NPF pathway, the EGFR/Erk and the PI3K/Act pathways have also been implicated in ethanol sedation (Wen et al., 2005 ; Corl et al., 2009 ; Eddison et al., 2011). More recently, it was found that the *Drosophila* dopamine/ecdysteroid receptor (DopEcR) mediates ethanol sedation by inhibiting EGFR/Erk signaling to promote ethanol sedation (Petruccelli et al., 2016). The GABA-B receptor, the *aru* gene, which encodes a homologous adaptor protein to mammalian Epidermal Growth Factor Receptor Substrate 8, the tumor suppressor homolog gene *tank*, and the *gfa* gene, a Dα7 nAChR subunit, have also been found to play a role in ethanol sedation (Dzitoyeva et al., 2003 ; Eddison et al., 2011 ; Devineni et al., 2013 ; Velazquez-Ulloa, 2017). *homer* function was needed in R2/R4 ellipsoid body neurons for ethanol sedation (Urizar et al., 2007). Corazonin neurons, which express the neuropeptide corazonin and the transcription factor *apontic (apt)* also modulate ethanol sedation (McClure and Heberlein, 2013). The autophagy gene *Atg16* acts in corazonin-expressing neurosecretory cells to regulate ethanol sedation, and seems to regulate corazonin transcript and protein levels (Varga et al., 2016).

Examination of gene expression on a microarray after 30 min of 60% ethanol vapor compared to flies exposed to water vapor identified several genes with altered expression in ethanol-exposed flies (Kong et al., 2010a). These genes had functions in serine biosynthesis, olfaction, transcriptional regulation, cytoskeletal organization, immunity and metabolism (Kong et al., 2010a). *Sir2* transcript and protein expression was greatly reduced after ethanol exposure (Morozova et al., 2006 ; Kong et al., 2010a ; Engel et al., 2016). Along with decreased expression, acetylation of Histone 3 at Lysine 9 (H3K9) was increased (Morozova et al., 2006 ; Engel et al., 2016). This is consistent with *Sir2*’s role as a deacetylase that targets H3K9 (Engel et al., 2016). *Sir2* mutants had decreased ethanol sedation sensitivity and ethanol sedation tolerance, and it was further showed that *Sir2* is required in adult mushroom body α/β lobe neurons for these effects (Engel et al., 2016). Synapsin (*Syn*) expression was greatly decreased in *Sir2* mutants and it was further shown that *Syn* expression decreased after ethanol exposure in wild-type but not in *Sir2* mutant flies (Engel et al., 2016). The protein levels of Syn were also decreased in ethanol treated brains (Engel et al., 2016). As expected, *Syn* mutants had decreased ethanol sensitivity and tolerance (Engel et al., 2016).

**FIGURE 6:**
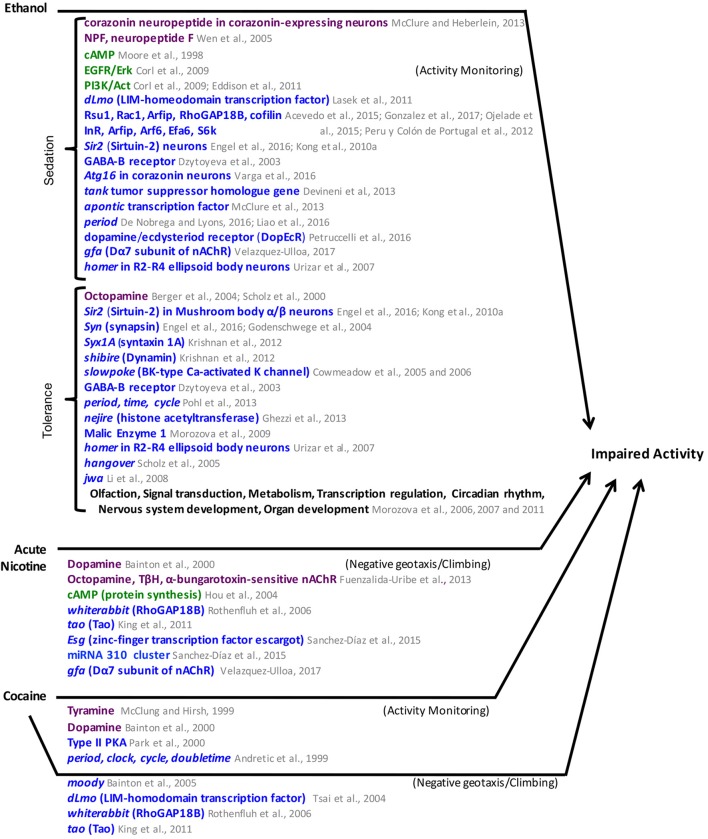
Mediators of ethanol, acute nicotine, and cocaine-induced impaired activity in *Drosophila*. Neuronal pathways (dark purple), cellular pathways (dark green), genes/proteins (royal blue). nAChR, nicotinic Acetylcholine receptor.

The results described above are summarized in **Figure 6**. Common factors in the mechanisms for ethanol hyperactivity and ethanol sedation include roles for RhoGAP18B, the EGFR pathway, and the gene *tao*. Common factors between ethanol sedation mechanisms and those of natural reward include roles for the cAMP and insulin pathways and the *Sir2* and *Syn* genes.

#### Ethanol Tolerance

Ethanol tolerance is the sedation response after a second ethanol exposure. The ethanol tolerance assay has identified several genes and molecular pathways in *Drosophila* that mediate this effect. The OA pathway was found to mediate ethanol tolerance (Scholz et al., 2000 ; Berger et al., 2004). The *hangover* gene (*hang*), a zinc finger protein, and *jwa*, which are genes involved in stress responses, mediate ethanol tolerance (Scholz et al., 2005 ; Li et al., 2008). The *slowpoke* gene (*slo*), which encodes a BK-type Ca-activated K channel, is also involved in ethanol tolerance (Cowmeadow et al., 2005, 2006). The GABA-B receptor and the gene *homer*, which interacts with metabotropic glutamate receptors on the post-synaptic site, have also been implicated in ethanol tolerance (Dzitoyeva et al., 2003 ; Urizar et al., 2007). Homer function was needed in R2/R4 ellipsoid body neurons for ethanol tolerance (Urizar et al., 2007). The pre-synaptic genes *synapsin*, *syntaxin 1A*, and *shibire* were also found to regulate ethanol tolerance (Godenschwege et al., 2004 ; Krishnan et al., 2012). *Sir2* mutants had reduced ethanol sedation tolerance (Kong et al., 2010a). The clock genes *per*, *tim*, and *cyc* also modulate ethanol tolerance (Pohl et al., 2013). More recently it has been shown that ethanol exposure results in the histone acetylation of genes that form a network for ethanol tolerance. The histone acetyltransferase that mediates these histone modifications is coded by the gene *nejire*, the *Drosophila* ortholog of mammalian CBP (Ghezzi et al., 2013).

A different approach taken to identify genes that regulate ethanol sensitivity and tolerance is to determine global changes in gene expression after ethanol exposure. In one study, transcript expression level was analyzed in flies exposed to ethanol during a postural control assay and again 2 h later (Morozova et al., 2006). This study identified downregulation of genes that function in olfaction and upregulation of signal transduction genes after a single ethanol exposure, and downregulation of metabolic enzymes, and upregulation of transcriptional regulators and circadian genes only after a second exposure to ethanol (Morozova et al., 2006). Another approach using artificial selection for ethanol sensitivity identified 32 mutants with significantly different responses to ethanol compared to their genetic control; 23 of these had human orthologs (Morozova et al., 2007). These genes were involved in carbohydrate metabolism, lipid metabolism, nervous system development, transcription regulation, and signal transduction (Morozova et al., 2007). Analysis of the variation in ethanol tolerance in 40 inbred lines with genome-wide variation in a gene expression study identified genetic networks that mediate this effect, including a network with Malic Enzyme 1 (Morozova et al., 2009). A new approach combined screening a co-isogenic P-element insertion mutant collection to identify lines with differential ethanol sensitivity, and then used computational approaches to build genetic networks based on transcription correlation from whole-genome expression data (Morozova et al., 2011). This approach identified focal genes in the networks that were validated as having a role in ethanol sensitivity in wild-type flies, and also validated that these genes worked in a single network (Morozova et al., 2011).

**Figure 6** summarizes the results of the studies above. Common genes that mediate ethanol tolerance and ethanol sedation include the GABA-B receptor, *homer*, *synapsin*, and *Sir2*. Whole genome analysis of gene expression after ethanol exposure identified metabolism genes among the genes regulated by ethanol exposure. It would be interesting to try a similar approach to identify gene expression changes after exposure to natural rewards. Future research could also examine whether some of the pathways involved in ethanol’s effects like Corazonin, EGFR/PI3K, or RhoGAP18 and cytoskeleton regulation also have roles in natural reward.

Ethanol has been studied more than other drugs but the current data shows similarities in the genes and pathways mediating the effects of ethanol and the drugs discussed below.

### Other Drugs

#### Cocaine

There are parallels between the results from these studies with research in mammals that suggest *Drosophila* is a viable model to study cocaine reward. Flies exhibit specific locomotor effects when exposed to cocaine and develop sensitization after repeated exposure (McClung and Hirsh, 1998). Several molecular pathways have been implicated in cocaine’s effects in the fly (Hirsh, 2001 ; Heberlein et al., 2009). Type II PKA activity mediates cocaine sensitization (Park et al., 2000). The dopaminergic pathway and tyramine also modulate cocaine sensitivity (McClung and Hirsh, 1999 ; Bainton et al., 2000). The *moody* gene, which encodes a G-protein -coupled receptor that regulates blood-brain-barrier permeability in flies, *whiterabbit* and *tao* also mediate cocaine sensitivity (Bainton et al., 2005 ; Rothenfluh et al., 2006 ; King et al., 2011). Cocaine sensitivity is also mediated by *dLmo* (Tsai et al., 2004). Mutant flies for several circadian genes fail to develop cocaine sensitization, including flies mutant for *period*, *clock*, *cycle*, and *doubletime* (Andretic et al., 1999). These circadian genes were first linked to cocaine sensitization in flies, and have now been linked to cocaine sensitization and reward in mammals (Abarca et al., 2002 ; McClung et al., 2005).

#### Amphetamines and Methamphetamine

There have not been any studies to determine whether amphetamines are rewarding in fruit flies. However, the acute locomotor effects of amphetamine have allowed for the identification of the conserved effects of amphetamines in flies. Amphetamine increases locomotion in *Drosophila* larvae (Pizzo et al., 2013). The effects of amphetamine are mediated by DA, DA transporter phosphorylation, and membrane raft protein Flotillin 1 (Pizzo et al., 2013). Another study found a contribution of PIP_2_ in mediating the locomotor effects of amphetamine in *Drosophila* (Hamilton et al., 2014). It was shown that the DA transporter associates with PIP_2_ in cell culture and that this interaction is needed for amphetamine-induced DA efflux and for amphetamine-induced locomotion in *Drosophila* (Hamilton et al., 2014). More recently it was shown that the *Drosophila* vesicular monoamine transporter (dVMAT) is also needed for the amphetamine-induced hyperlocomotion in fruit flies (Freyberg et al., 2016). Flies with a null mutation in dVMAT did not develop amphetamine-induced hyperlocomotion (Freyberg et al., 2016).

Methamphetamine has also been shown to increase locomotion in adult flies through Rab10, a GTP-binding protein present in membrane rafts that regulates intracellular membrane trafficking (Vanderwerf et al., 2015). Rab10’s abundance within rafts is decreased after methamphetamine exposure (Vanderwerf et al., 2015). Flies with a mutant form of Rab10 had decreased sensitivity to methamphetamine-induced increased locomotion and needed a larger methamphetamine dose to display significantly increased locomotion compared to the controls (Vanderwerf et al., 2015). Other proteins whose abundance was affected by methamphetamine exposure included the microtubule-associated protein 1A, the NAD-dependent histone deacetylase Sirtuin-2, and the Rho-related GTP-binding protein Rho G (Vanderwerf et al., 2015).

#### Nicotine

Nicotine reward has not been established in flies. However, probing the acute effects of nicotine has revealed molecular mechanisms similar to cocaine (Bainton et al., 2000). The dopaminergic pathway, which modulates nicotine sensitivity, was tested in a climbing assay based on flies’ natural behavior of negative geotaxis (Bainton et al., 2000). Flies acutely exposed to nicotine became unable to climb, but this effect was reduced when DA was depleted (Bainton et al., 2000). OA was also shown to mediate nicotine’s effects on a similar assay, as flies with decreased OA were not affected by nicotine (Fuenzalida-Uribe et al., 2013). OA release is mediated by the activation of α-bungarotoxin -sensitive nAChRs in the brain (Fuenzalida-Uribe et al., 2013).

The cAMP pathway mediates the effect of nicotine on negative geotaxis (Hou et al., 2004). Flies with increased levels of cAMP were more sensitive to nicotine’s effects in the climbing assay, and flies with mutations in PKA were less sensitive to the effects of nicotine (Hou et al., 2004). Repeated exposure to nicotine in adult flies increased the effect of nicotine on the flies’ ability to climb when tested at 4, 8, and 20 h after the first nicotine exposure (Hou et al., 2004). The sensitization of the response to nicotine is mediated by the cAMP pathway, including *dCREB*, and requires protein expression (Hou et al., 2004).

Additional genetic mechanisms mediating nicotine’s effects in the climbing assay have been uncovered. Flies with mutations in the *whiterabbit* or in *tao* have decreased sensitivity to nicotine in a negative geotaxis climbing assay (Rothenfluh et al., 2006 ; King et al., 2011). A genetic screen identified two mutant lines with increased sensitivity to nicotine that had significantly longer recovery times after nicotine exposure (Sanchez-Díaz et al., 2015). The mutations mapped onto the transcription factor gene *escargot* (*esg*) and the miRNA 310 cluster (Sanchez-Díaz et al., 2015).

A different study characterized the effects of chronic nicotine exposure in adult flies and found that flies became hyperactive (Ren et al., 2012). This study identified *Dcp2*, the gene encoding the decapping protein 2, as a mediator of this chronic nicotine-induced locomotor hyperactivity (Ren et al., 2012). This study also identified the *gfa* gene, which encodes for the Dα7 nAChR subunit, as a mediator of chronic nicotine-induced locomotor hyperactivity, as flies with downregulated Dα7 did not develop hyperactivity (Ren et al., 2012).

The studies described above focus on the effects of nicotine exposure in adult flies. Developmental nicotine exposure in *Drosophila* also affects how exposed flies respond to nicotine when they are adults (Velazquez-Ulloa, 2017). After developmental nicotine exposure, flies display decreased sensitivity to acute nicotine exposure in the climbing assay. They also display decreased sensitivity to ethanol as adults in an ethanol sedation assay (Velazquez-Ulloa, 2017). In addition, developmental nicotine exposure resulted in decreased survival, increased developmental time and decreased weight (Velazquez-Ulloa, 2017). The nAChR subunit Dα7 mediated the effects of developmental nicotine on survival and developmental time, and may also mediate the effects on nicotine sensitivity (Velazquez-Ulloa, 2017). Different studies examining genetic variation associated with larval resistance to nicotine on a survival assay using the *Drosophila* Synthetic Population Resource identified *Ugt86Dd* as a locus that confers differential sensitivity to nicotine (Marriage et al., 2014 ; Highfill et al., 2017).

These studies identify DA as a common mediator of drug effects. Other neurotransmitters have been shown to play a role in the effects of specific drugs. More testing is needed to determine if these neurotransmitters mediate responses to most drugs. RhoGAP18B along with proteins both upstream and downstream, mediate effects of ethanol. These pathways regulate cytoskeleton dynamics. It will be interesting to determine the involvement of these pathways in mediating the effects of other drugs. Circadian genes and genes that encode proteins that modify histones are also common factor mediators of drug effects that warrant additional investigation (**Figures 5**, **6**).

## Perspective on the Common Mechanisms of Natural and Drug Reward

Analysis of the scientific literature included here suggests that there are parallel circuits mediating perception and reward for each appetitive natural stimulus. Sensory receptors in the periphery are activated by different taste modalities. This sensory information is conveyed to different neuronal circuits in the *Drosophila* central brain, including the activation of specific subsets of dopaminergic neurons that connect to distinct mushroom body compartments that encode either short or long-term memories. Memory formation requires the cAMP pathway in mushroom body neurons to mediate the synaptic plasticity for encoding memories. These natural reward memories are homeostatically modulated by hunger and thirst. Serotonin and NPF convey nutrient signals via activation of PPL1 MB-MP1 neurons, which have calcium oscillations that are modulated by hunger state and represented by sugar levels in the hemolymph. The receptors, dopaminergic neurons, and mushroom body compartments have been determined for sugar and water reward, but have not yet been identified for protein or fatty acid reward. The neural circuits that mediate conditioned odor preference and oviposition preference for ethanol are remarkably similar to those for sugar and water reward, but seem to be a parallel circuit. More detailed mapping of ethanol reward circuits will determine if there is overlap between ethanol, sugar and other rewards.

Palatability and preference for different nutrients and ethanol also have common factors. Serotonin plays a role in both sugar and protein preference, while the cAMP pathway plays a role in sugar and ethanol consumption preference. Several neuropeptides mediate nutrient preference including insulin, juvenile hormone inducible 21, and Dh44. Dh44-expressing neurons mediate preference for a nutritious sugar, and similar to PPL1 MB-MP1 neurons, exhibit calcium oscillations that are modulated by glucose levels in the hemolymph. Hence, calcium oscillation modulation by nutrient levels in the hemolymph seems to be a common mechanism for encoding hunger.

The study of natural reward in *Drosophila* has developed around testing the reinforcing properties of stimuli that lead to either appetitive or aversive memories and mapping the neural circuits underlying these memories with continuously improving resolution. The study of drug reward began by focusing on acute effects of drugs and then identifying the genes that mediated the acute effects. More work needs to be done to map where the genes, proteins and signaling cascades function in the neural circuits that mediate drug reward. In addition, it would be interesting to test whether genes and signaling pathways that mediate drug effects also have roles on natural reward. Of particular interest are the signaling cascades with RhoGAP18B at the center that involve cytoskeleton dynamics, genes involved in development of reward brain centers such as *tao*, circadian genes, and histone modification genes such as *Sir2* and *nejire*.

A model is emerging for parallel circuits for reward from sensory perception to behavior segregated by the type of stimulus. The reward system is centered around dopaminergic neurons as carriers of the reinforcement signal with the mushroom body as coincidence detector center, where integration of information occurs at specific compartments of the mushroom body, which in turn recruit different sets of mushroom body output neurons (de Araujo, 2016 ; Scaplen and Kaun, 2016 ; Kaun and Rothenfluh, 2017 ; Cognigni et al., 2018).

Future studies of drug reward with assays that focus on the reinforcing properties of the drugs instead of just the acute effects will make it possible to determine the similarities and differences in the encoding of natural and drug reward in *D. melanogaster*. The unparalleled genetic and molecular tools available for *Drosophila* research will continue to allow for the mapping of neuronal circuits at single-cell resolution. Combining this approach with the ability to manipulate genes in individual cells makes *Drosophila* an ideal model organism to dissect the mechanisms of both natural and drug reward.

## Author Contributions

NV-U wrote the manuscript draft and provided the feedback on the figures. EL wrote the sections of the manuscript, edited the full draft, and prepared the figures and all other sections of the paper.

## Conflict of Interest Statement

The authors declare that the research was conducted in the absence of any commercial or financial relationships that could be construed as a potential conflict of interest.

## Abbreviations

AC: adelylyl cyclase
CS: conditioned stimulus
DA: dopamine
MB: mushroom bodies
MNC: median neurosecretory cells
nAChR: nicotinic acetylcholine receptor
NPF: neuropeptide F
OA: octopamine
PAM: protocerebral anterior medial
PI: pars intercerebralis
PPL1: protocerebral posterior lateral
PPM3: protocerebral posterior medial
SOG: subesophageal ganglion
TβH: tyramine beta hydroxylase
US: unconditioned stimulus
